# Self-reported healthcare waste segregation practice and its correlate among healthcare workers in hospitals of Southeast Ethiopia

**DOI:** 10.1186/s12913-019-4439-9

**Published:** 2019-08-22

**Authors:** Biniyam Sahiledengle

**Affiliations:** Department of Public Health, Madda Walabu University Goba Referral Hospital, P.o.box: 76, Bale, Goba, Ethiopia

**Keywords:** Healthcare waste, Biomedical wastes, Sharps, Infectious waste, Hospitals, Segregation, Waste management, Ethiopia

## Abstract

**Background:**

The key to the effective management of healthcare wastes is segregation of the waste at the point of generation; no matter what final strategy for treatment and disposal of wastes is selected, it is critical that waste streams are separated. In Ethiopia, healthcare waste segregation practice among healthcare workers is overlooked and scarcely addressed in the scientific literature. This hospital-based cross-sectional study was, therefore, conducted to assess healthcare waste segregation practice and its correlate among healthcare workers in Bale zone, southeast Ethiopia.

**Methods:**

All five hospitals found in Bale zone were included and the study participants were selected using a systematic sampling technique from each hospital. Data were collected through interview using structured questionnaires. Descriptive statistics were computed. Bivariate and multivariable logistic regression analyses were employed to identify factors that correlate with healthcare waste segregation practice.

**Results:**

A total of four hundred and nine healthcare workers participated in the study, for a response rate of 97.4%. Of these, 220(53.8%) (95% CI: 49.1–58.9) of healthcare workers were found to have reported good healthcare waste segregation practice. Being male gender (AOR = 1.70, 95%CI: 1.04–2.78), less than 30 years of age (AOR = 2.02, 95%CI: 1.06–3.84), less than 2 years work experience (AOR = 2.95, 95%CI: 1.39–6.26), having good self-reported standard precaution practice (AOR = 8.47,95%CI:4.98–14.42), and working in a department with an on-site healthcare waste segregation container (AOR = 2.10, 95%CI:1.24–3.55) were factors that correlated with self-reported healthcare waste segregation practice.

**Conclusion:**

Overall, only half of the healthcare workers had good healthcare waste segregation practice, which is low and unsatisfactory. Less service year, having good standard precaution practice, and the presence of onsite waste segregation container were the most important variables that correlate with self-reported healthcare waste segregation practice. Therefore, to improve healthcare waste segregation practice health authorities should focus on sufficient allocation of onsite waste receptacles. In addition, periodic training on standard precaution will improve compliance with segregation practice.

**Electronic supplementary material:**

The online version of this article (10.1186/s12913-019-4439-9) contains supplementary material, which is available to authorized users.

## Background

In performing healthcare activities, healthcare facilities (HCFs) generate healthcare waste (HCW) that could be potentially harmful to healthcare workers, the public and the environment [1]. Injuries, the transmission of infections, environmental pollution, fire hazards, and public nuisances (offensive smells, unsightly debris, etc.) are the major risks and hazards of poorly managed HCW [2, 3]. Hence, management of healthcare wastes requires special attention and needs to be assigned high priority [4–6].

Safe healthcare waste management (HCWM) practices reflect the quality of the services in any HCF, and it includes all activates of waste generation, segregation, transportation, storage, treatment and disposal [7, 8]. The key to minimization and effective management of HCW is segregation of the waste at the point of generation; no matter what final strategy for treatment and disposal of wastes is selected, it is critical that waste streams are separated to protect both humans and the environment [9, 10].

Segregation means separating different wastes into different color-coded bins with liners or sharps containers at locations where they are generated, and it is always the first and the most important activity in HCWM [3, 11]. The absence of proper HCW segregation increases the risk of occupational injury and blood born viral infections, particularly among waste handlers, as a result, waste handlers should never sort through waste after it has been placed in a bin [3, 11]. To overcome such problem, the Federal Ministry of Health of Ethiopia recommended color coding waste segregation practice in all healthcare facilities. The recommended color coding scheme is a black bin with liner for non-infectious wastes, a yellow bin liner with biohazard symbol for infectious wastes, a red bin liner with biohazard symbol for pathological and anatomical wastes, a brown bin with liner for chemicals wastes, yellow bin with radioactive label for radioactive wastes, and yellow box marked “SHARPS” with biohazard symbol for sharps [3].

The World Health Organization (WHO) estimated, around 75–90% of the waste produced by hospitals are general or non-hazardous wastes comparable to domestic wastes, while the remaining 10–25% is regarded as hazardous and may impose risks due to infectious, pathological, chemical and radioactive materials or sharps [9]. In most cases this proportion can be achieved by proper segregation of waste streams; if the infectious component is mixed with the general waste stream, the entire mass becomes potentially infectious [11].

Currently, in many developing countries, poor segregation and the question of how to manage HCWs has become a critical concern [2, 11, 12]. The problem got particular attention back in 2002 by the WHO. The study conducted by WHO in 22 developing countries showed that the proportion of facilities that did not manage waste properly and used inappropriate waste disposal methods ranged between 18 and 64% [12]. Similarly, other recent studies also reported the quantity of HCWs has risen sharply in recent years accompanied by inadequate HCWM [13–19]. Moreover, studies from Greece and Brazil demonstrate that inappropriate segregation practice leads to an increase in the amount of infectious waste generation [17, 18].

In Ethiopia, as in many developing countries, compliance with the recommended HCW segregation practice still not jumped from paper [4, 6, 16]. Moreover, safe HCWM has been given very little attention and many facilities do not meet the minimum standards required for proper handling of HCWs [7, 16]. The previously conducted studies showed that the proportion of HCW generation is significantly higher than the WHO threshold; the WHO threshold is 80% general HCW, 15% pathological and infectious waste, 1% sharps waste, 3% chemical or pharmaceutical waste, and less than 1% special waste, such as radioactive or cytostatic waste, pressurized containers or broken thermometers and used batteries [19–24]. For example, a study conducted in six hospitals of Addis Ababa (Ethiopia) shows that the proportion of hazardous HCW ranges from 29.5 to 53.12% [21]. In Menellik II hospital (Ethiopia), the proportion of infectious waste was 53.73% [25]. In the north and south Ethiopia, the proportions of infectious wastes in hospitals were 34.3 and 53%, respectively [19, 26]. In general, these figures are about three to four times greater than the threshold value recommended by WHO [9]. The foremost explanation for the different estimates regarding the share of general and hazardous constituents of HCW generation may be due to the possibility that segregation of hospital waste streams is weak [16, 22, 27, 28]. In addition, a lack of enforced public health regulations for HCWs segregation may exacerbate the current situation [16, 27, 29].

Poor segregation of HCWs can result in additional costs related to HCW disposal and poses various environmental and public health threats [18, 30]. However, proper segregation of HCWs should result in a clean solid waste stream which can be easily, safely and cost-effectively managed through recycling, composting and landfilling [10]. The volume of waste in the overall HCWs stream could be reduced by as much as 60% through careful segregation of items [31]. In this regard, understanding the underlying factors associated with HCW segregation behaviors is a vital step towards developing interventions to improve the waste management system [32]. Therefore, the aim of this study was to determine HCW segregation practice and to identify factors that correlate with HCW segregation practice among healthcare workers in hospitals of Bale zone, southeast Ethiopia.

## Methods

### Study design and setting

A hospital-based cross-sectional study was done from March 1 to 28, 2018. The study includes all hospitals found in Bale zone (namely Goba referral hospital, Ginnir general hospital, Robe general hospital, Dellomena general hospital and Madda Walabu primary hospital) in southeast Ethiopia. Bale zone is located 445 km away from Addis Ababa, the capital city of Ethiopia. At the time of this study, there were a total of 758 fulltime healthcare workers working in all hospitals, according to Bale zone human resources 2017 annual report.

### Study participants

The source population of the study includes all healthcare workers found in five Bale zone hospitals. The study populations were all selected healthcare workers in five Bale zone hospitals. All healthcare workers who have the qualification of medical doctors, health officers (health officers are trained with the knowledge and skills that are required to solve and manage the common clinical disorders and the potentially preventable public health problems in primary health care settings, such as district hospital and health center), nurses, midwives, and laboratory technician/technologist who work at least 6 months in the care of patient were included in the study. Healthcare workers who work at least 6 months and above included, since in Ethiopian public healthcare facilities 6 month is the minimum trial period for any healthcare professional before he/she accepted as full employed healthcare workers. Healthcare workers who were on maternal leave during the data collection period were excluded (four healthcare workers were excluded since there are on maternity leave and two healthcare workers were on annual leave).

### Sample size determination and sampling procedure

The sample size was determined using Epi Info™ 7.1.1.14 statistical software (Center for Disease Control and Prevention, 2013) using single population proportion formula with the assumption of 95% confidence level, 5% precision and considering the proportion of healthcare workers who correctly practiced HCWs segregation was 46.3% in Gondar university hospital [30], and considering a possible non-response rate of 10%. The calculated sample size was (*n* = 420).

First, the calculated sample size (*n* = 420) was distributed to each Bale zone hospitals proportional to the size of healthcare workers. Thereafter, a systematic sampling technique was employed to select health care workers from the sampling frame (Additional file 1: The schematic presentation of sampling procedure).

### Variables and measurement

The dependent variable of the study was healthcare workers self-reported HCW segregation practice. HCW segregation practice was measured by enquiring whether the respondent had practiced the recommended HCW segregation practice in their workplace using eight (yes/no) questions, each correct segregation practices was awarded one point and if not zero. Afterward, the total practice score of the respondent was calculated and summed up to give the overall practice. Then, healthcare workers who had scored six and above value (≥ 75%) of the cumulative score on HCWs segregation questions were labeled as “good practice” if not “poor practice”. According the Federal Ministry of Health infection prevention and patient safety guideline recommendation for HCWM practice, the minimum HCWs segregation technique in Ethiopia is to segregate HCWs into three compartments (general, infectious and sharp wastes). Following this recommendation, the study considered those three HCWs segregation practice and other interconnected activities, as a result a score of 6 and above value (out of 8 items that ≥75%) was determined as a cut of point.

The independent variables includes socio-demographic factors (sex, age, marital status, years of service, educational status, and profession), institutional factors (availability of on-site waste collection container, training about HCWM, presence of guideline/standard operating procedure (SOP) or instructive poster on HCW segregation) and individual related variables (standard precaution practice, awareness on the different categories of HCW, awareness on safe management of HCW, and attitude towards HCW segregation.

To determine healthcare workers standard precautions practice; the respondents were asked ten questions to assess their overall standard precautions practice. Each correct standard precaution practice was awarded one point, otherwise zero. Afterward, a composite score was constructed and healthcare workers standard precaution practice was classified as good (if equal to and above the mean) and poor (below the mean). The mean was considered as a cut of point seeing the result was normally distributed (Additional file 2).

Attitude towards HCW segregation variables comprised of 6 statements with response categories “agree”, “disagree” or “neutral”. Composite scores were calculated and those scored equal to and above the mean value for the composite score of attitude questions were labeled as having “favorable attitude” towards HCWs segregation, if not “unfavorable attitude” [30].

### Data collection and quality

An interviewer administrated a pre-tested structured questionnaire was used for data collection. The data collection tool was developed by the author considering the national healthcare waste management guideline [33] and related kinds of literature [3, 7, 9, 34] (Additional file 3**)**. The data collection tool was first developed in English and translated to Amharic (local language) then back-translated to English in order to assure consistency of the questions. Pre-testing was done in 21 healthcare workers out of the study area to reduce measurement bias. The questionnaire was also tested for internal consistency using Cronbach’s alpha test and a score of 0.791 and 0.835 was obtained for healthcare waste segregation and standard precaution practice questionnaires, respectively.

Five trained BSc nurses (who were from another healthcare facility that were not included in the study) collected data through a face-to-face interview. To enhance the quality of data one-day training was given for data collectors and supervisors regarding the aim of the study, data collection procedure, and data collection tool. Before data collection, all participants were fully informed regarding the objective of the study, and informed consent was obtained from each study participant. The collected data were treated as confidential. The completeness and consistency of the questionnaire were checked by two supervisors and by principal investigator throughout the data collection period.

### Statistical analysis

Data analysis was undertaken using SPSS version (20.0). Descriptive statistics such as frequencies, mean and standard deviation were computed. Binary and multivariable logistic regression models were employed to identify factors associated with self-reported HCW segregation practice. All the independent variables were tested for possible multicollinearity before putting those into the multivariable logistic regression models. And variables with a *p*-value of less than 0.25 in the bivariate analysis were then entered into a multivariable logistic regression to control the effect of confounders [35]. Adjusted Odds ratios (AOR) with corresponding 95% confidence interval (CI) were estimated to assess the strength of association and for all statistically significant tests *p*-value < 0.05 was used a cut-off point. The overall goodness of fit was checked using the Hosmer and Lemeshow test.

## Results

### Socio-demographic and other characteristics of healthcare workers

A total of 409 healthcare workers participated in the study for a response rate of 97.4%. Among these participants 210 (51.3%) healthcare workers were males and 199(48.7%) were females, with a male to female ratio of 1.1: 1. The mean (standard deviation (SD)) age of HCWs were 28.12 (± 5.18), and 176(43.0%) had more than five-year work experience (Table 1).

**Table 1:** Socio-demographic, individual and health facility related variables of healthcare workers in hospitals of Bale zone, Southeast Ethiopia March 2018 (*n* = 409)

Variables	Category	Frequency	%
Sex			
	Male	210	51.3
	Female	199	48.7
Age (years)			
	< 25	80	19.5
	25–29	213	52.1
	30–34	83	20.3
	≥35	33	8.1
Marital status			
	Married	221	54.0
	Single	188	46.0
Years of service (years)			
	< 2 years	116	28.4
	2–5 years	117	28.6
	> 5 years	176	43.0
Hospital type			
	Referral	159	38.9
	General	218	53.3
	Primary	32	7.8
Current working department			
	Internal medicine	69	16.9
	Surgical ward	55	13.4
	Pediatrics ward	33	8.1
	Neonatal intensive care unit (NICU)	23	5.6
	Obstetrics and Gynecology	98	23.9
	Operating room (OR)	21	5.1
	Emergency-unit	29	7.1
	Outpatient department (OPD)	45	11.1
	Laboratory and others^a^	36	8.8
Profession			
	Nurses and midwifery	254	62.1
	Physician and health officer	57	13.9
	Laboratory technicians and technologist	98	24.0
Educational status			
	First degree and above	228	55.7
	Diploma	181	44.3
Presence of guideline, SOP or instructive poster on HCW segregation			
	Yes	237	57.9
	No	172	42.1
Ever taking training in HCWM methods			
	Yes	53	13.0
	No	356	87.0
Know the different categories of HCWs			
	Yes	322	78.7
	No	87	21.3
Awareness on HCW segregation			
	Yes	266	65.0
	No	143	35.0
Standard precaution practice			
	Good	215	52.6
	Poor	194	47.4
Attitude towards HCW segregation			
	Favorable	249	60.9
	Unfavorable	160	39.1

^a^Triage, dental clinic, Eye clinic, ART clinic, TB-clinic, Maternal and child health unit

Of the total respondents, only 53(13.0%) of healthcare workers received training related to HCWM in past 1 year preceding the study period. Regarding healthcare workers knowledge on the different types of healthcare wastes, 322 (78.7%) of healthcare workers correctly know the different types of HCWs, such as sharps, infectious and general wastes. Two hundred and sixty-six (65.0%) of the respondent had awareness of HCWs segregation by type.

In addition, standard precautions practice of healthcare workers were assessed for the main components like (proper use of personal protective equipment, hand hygiene practice, safe injection practice, preventing of nosocomial infection, and waste management practice) and 215(52.6%) [95%CI: 47.4–57.0] had good standard precautions practice and 249 (60.9%) [95%CI: 56.2–65.5] had a positive attitude towards HCW segregation (Table 1).

### Self-reported healthcare waste segregation practice

Two hundred and twenty (53.8%) [95% CI: 49.1–58.9] healthcare workers had self-reported good HCWs segregation practice. And 139 (33.9%) of healthcare workers only segregate HCW for their last client.

### Observational results on HCW treatment and disposal practice

**Fig. 1:**
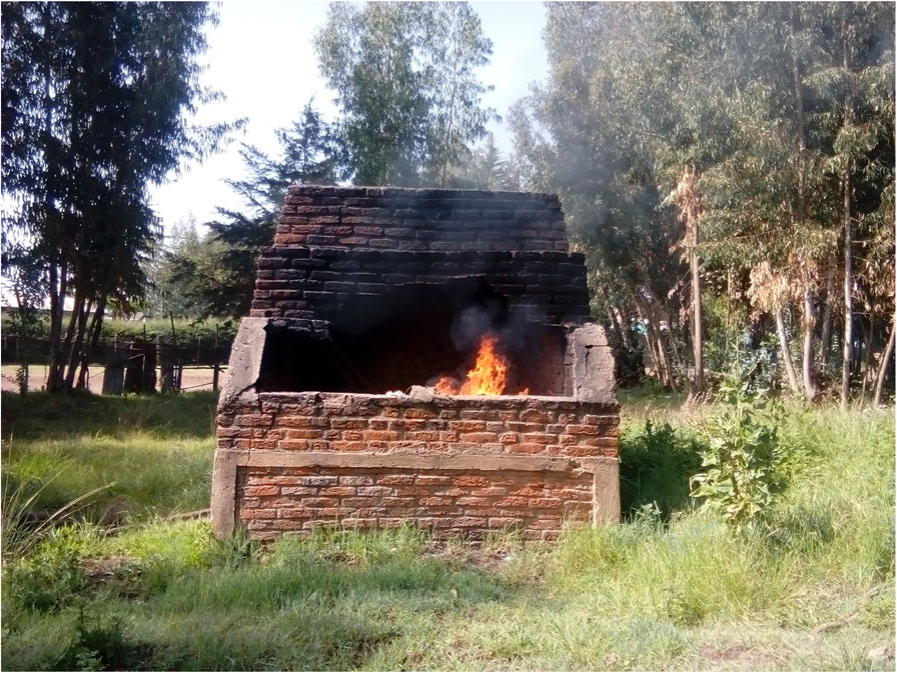
Poor condition of a brick incinerator at Bale zone hospitals, Southeast Ethiopia, 2018

**Fig. 2:**
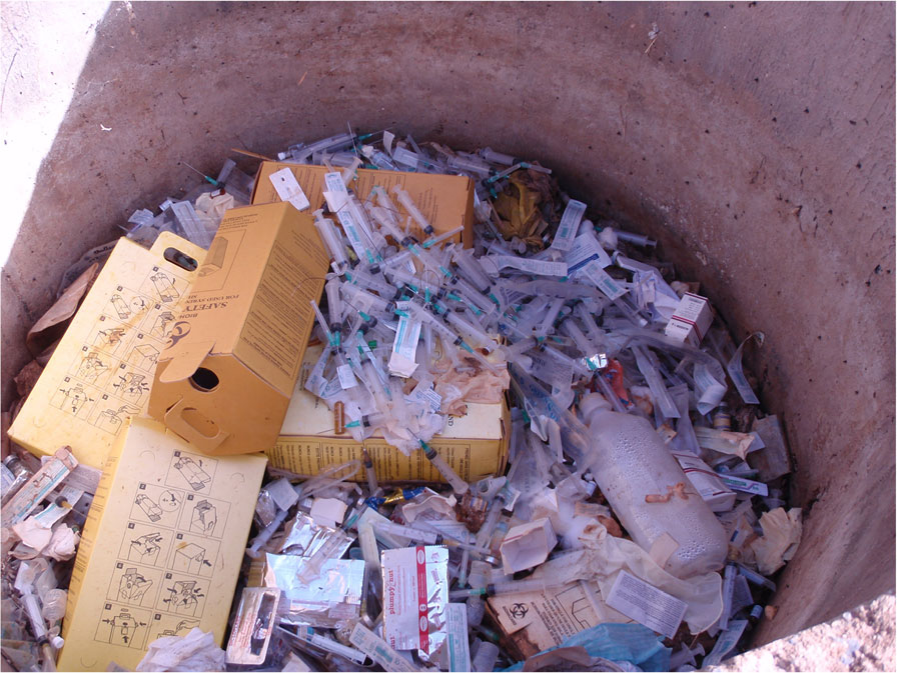
Indiscriminate disposal of sharp wastes at Bale zone hospitals, Southeast Ethiopia, 2018

**Fig. 3:**
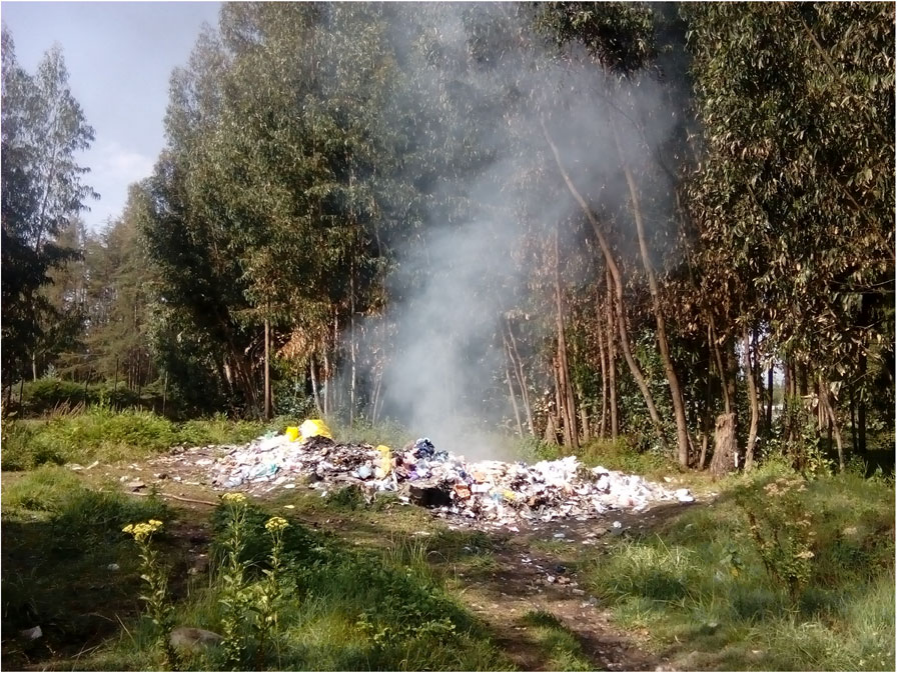
Open air burning of healthcare wastes at Bale zone hospitals, Southeast Ethiopia, 2018

**Fig. 4:**
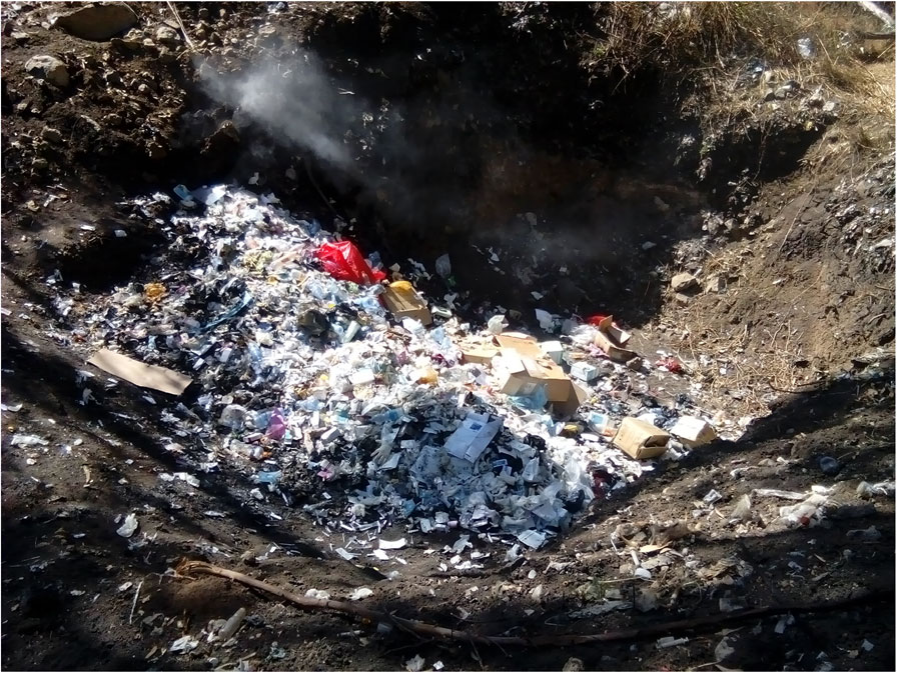
Open damping of healthcare wastes at Bale zone hospitals, Southeast Ethiopia, 2018

**Fig. 5:**
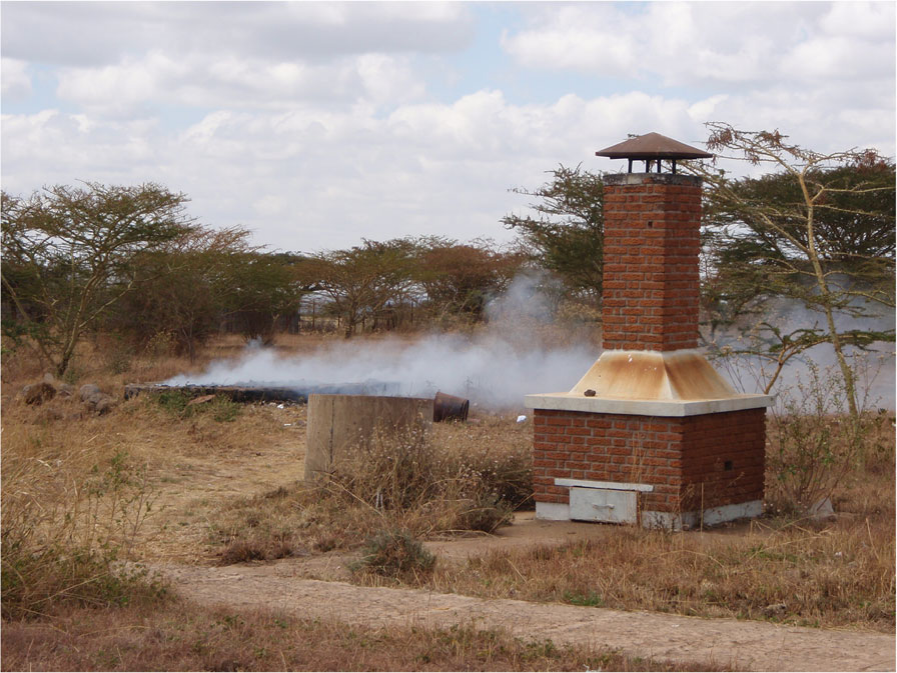
Uncontrolled and open air burning of healthcare wastes at Bale zone hospitals, Southeast Ethiopia, 2018

Table 2 shows the result of the observational assessment. From the empirical observation; all hospital treated and disposed of there HCW on-site. And all hospital practiced open pit burning of HCW. Even though, all hospitals have brick incinerator it was witnessed that all incinerator have some form of problem in terms of designing and construction. With respect to the disposal of the treated HCWs, all hospitals used open damping of HCWs in their compound (Figs. 1, 2, 3, 4 and 5).

**Table 2:** Observational assessment of healthcare waste treatment and disposal practices in Bale zone hospitals, Southeast Ethiopia 2018

Healthcare waste treatment and disposal practice description (*n* = 5)^a^	Yes
A designated area for waste treatment and disposal	5
Waste disposal site fenced	1
Having walkway to waste disposal site	3
On-site treatment of HCW practiced	5
On-site disposal of HCW	5
Presence of incinerators	5
Incinerators with some form of problems related to design and construction witnessed	5
Incinerators had remnants of incompletely burned HCW witnessed at the time of observational assessment	3
Presence of safe burial	0
Open pit/ open air/ burning and damping of HCW	5
Presence of placental disposal pit	5
Properly constructed watertight ash/needle pit witnessed	0
Offsite disposal (outsourcing) of HCW	0

^a^Number of hospitals

### Factors associated with healthcare waste segregation practice

In the bivariate analysis factors which were significantly associated with good self-reported HCWs segregation practice were: age, year of service, hospital type, current working department, profession, ever taking training in HCWM methods, awareness on HCW segregation, awareness on the different categories of HCWs, standard precaution practice and presence of on-site HCW segregation containers. After controlling the confounding in multivariable logistic regression analysis; gender, age, year of service, profession, standard precaution practice and presence of on-site HCW segregation containers were found to be significantly associated with good self-reported HCWs segregation practice.

To check the correctness of the final model, the Hosmer and Lemeshow test for the overall goodness of fit was used, and a value of 0.381 was obtained; that is not significant, which means the final model was correct. The result of the final model showed that male healthcare workers were 1.7 times more likely to had good self-reported HCW segregation practices than female healthcare workers (AOR = 1.70, 95% CI: 1.04–2.78). Those healthcare workers who are less than 30 years old were about 2 times more likely to had good self-reported HCW segregation practice than those who are 30 years or older (AOR = 2.02, 95% CI: 1.06–3.84). The study further identified those healthcare workers who served less than 2 years were about three times more likely to had good self-reported HCW segregation practice than those healthcare workers with greater than 5 years work experience (AOR = 2.95, 95% CI: 1.39–6.26).

In this study, physicians and health officers were 55% times less likely to had good self-reported HCW segregation practice than nurses and midwives (AOR = 0.45, 95% CI:0.21–0.98). Additionally, laboratory technicians/technologists were about 2.8 times more likely to had good self-reported HCW segregation practice than nurses and midwives (AOR = 2.80, 95% CI:1.49–5.26). The study also revealed that healthcare workers who had good self-reported standard precaution practices were about eight times more likely to had good self-reported HCW segregation practice than their counterpart (AOR = 8.47, 95% CI:4.98–14.42). Furthermore, those healthcare workers who were working in the department having on-site HCW segregation containers were about 2.1 times more likely to had good self-reported HCW segregation practice than their counterpart (AOR = 2.10, 95% CI:1.24–3.55) (Table 3).

**Table 3:** Factors associated with self-reported HCW segregation practice among healthcare workers in Bale zone hospitals, Southeast Ethiopia 2018

Variables	HCW segregation practice		Crude OR (95% CI)	Adjusted OR (95% CI)
	Good (*n* = 220)	Poor (*n* = 189)		
Sex				
Male	122	88	1.43 (0.97–2.11)	1.70 (1.04–2.78)**
Female	98	101	1	1
Age				
<30	170	123	1.82 (1.18–2.81)*	2.02 (1.06–3.84)**
≥30	50	66	1	1
Year of service				
<2 years	67	49	1.53 (0.95–2.46)	2.95 (1.39–6.26)**
2–5 years	70	47	1.67 (1.03–2.68)*	1.76 (0.91–3.43)
>5 years	83	93	1	1
Hospital type				
Referral	74	85	1	
General	124	94	1.51 (1.01–2.29)*	
Primary	22	10	2.53 (1.12–5.68)*	
Current working department				
Internal medicine and surgical	62	62	1	
Pediatrics ward and NICU	25	31	0.81 (0.43–1.52)	
Obstetrics and Gynecology	58	61	0.95 (0.58–1.57)	
The emergency unit, OPD, Laboratory, and others*	75	35	2.14 (1.26–3.65)*	
Profession				
Nurses and midwives	135	119	1	1
Physicians and health officers	22	35	0.55 (0.31–0.99)*	0.45 (0.21–0.98)**
Laboratory technicians and technologist	63	35	1.59 (0.98–2.56)	2.80 (1.49–5.26)**
Educational status				
First degree and above	128	100	1	
Diploma	92	89	0.81 (0.55–1.19)	
Presence of guideline, SOP or instructive poster on HCW segregation				
Yes	129	108	1.29 (0.86–1.94)	
No	91	81	1	
Ever taking training in HCWM methods				
Yes	37	16	2.19 (1.17–4.07)*	1.96 (0.95–4.05)
No	183	173	1	1
Awareness on HCW segregation				
Yes	155	111	1.68 (1.11–2.52)*	
No	65	78	1	
Awareness of the different categories of HCW				
Yes	162	160	1.97 (1.20–3.25)*	
No	58	29	1	
Standard precaution practice				
Good	155	60	5.13 (3.36–7.82)*	8.47 (4.98–14.42)**
Poor	65	129	1	1
Presence of on-site HCW segregation containers				
Yes	156	111	1.71 (1.34–2.58)*	2.10 (1.24–3.55)**
No	64	78	1	1
Attitude towards waste segregation				
Favorable	137	112	1.14 (0.76–1.69)	
Unfavorable	83	77	1	

* *p* < 0.05 crude; ** *p* < 0.05 adjusted; *OR* Odds Ratio, *CI* Confidence Interval

## Discussion

Healthcare waste that generated in the course of healthcare activities poses a significant health problem and must be managed accordingly, and the key to the effective management of HCWs is segregation of the wastes at the point of generation [10]. This study aimed to determine HCW segregation practice and its correlate among healthcare workers. The finding from this study suggest that 53.8% of the healthcare workers had good HCW segregation practice; put general, infectious, and sharp wastes into a different waste collection container. Factors such as gender of healthcare worker, age, service year, standard precaution practice, and presence of site waste segregation container were the most important variables that correlate with self-reported healthcare waste segregation practice.

Findings from this study show that a significant number of healthcare workers had poor HCW segregation practice. This finding is similar to studies conducted elsewhere in Ethiopia. For example, a study from Gondar town (north Ethiopia) reported HCW segregation practices was 31.9 and 46.3% [28, 30]. In addition, different related studies reported HCWM practices in Ethiopia remains a great challenge due to poor HCW segregation practice [4, 5, 24, 29, 36]. The present and the previously conducted studies suggested that HCW segregation practice requires special attention, and if the segregation process is poor or even a very small amount of hazardous waste is added to the general waste category, then the entire mass of the general waste can be unnecessarily polluted by the hazardous waste [16]. In line with this, several studies conducted in Uganda [32], Sudan [37], Iran [38], Jordan [39], Nigeria [40], and China [41] reported poor HCW segregation practice among healthcare workers.

In the Ethiopian context, multiple factors contribute to poor HCW segregation practice. The first reason may be due to the lack of separate regulation specific for the HCFs to enforce them for the proper management of the hazardous waste. In addition, a systematic review identified that lack of training, lack of awareness, staff resistance, managerial poor commitment, lack of adequate resources, negligence, and unfavorable attitude towards HCWM were the commonly identified challenges associated with poor HCWM in Ethiopia [16]. A study from Kenya also identified that poor compliance towards HCWM policy is one of the key reason for poor HCW segregation practice [42].

Interestingly, Hagen et al. [43] found that providing instructive posters as a tool to promote effective segregation of HCW appear to be a positive effect on HCW segregation practice among healthcare workers. However, in this study, the presence of instructive poster on HCW segregation practice was not found to be statistically significant. But, the crude odds ratio suggests that the presence of instructive tools may be associated with an increased odds of good HCW segregation practice. A study from Brazil also reported a similar finding [44].

In this study, healthcare workers who work in the department having on-site HCW segregation containers were more likely to have good HCW segregation practiced. This finding was in line with a study conducted in Gondar University (Ethiopia) [30]. The finding suggested that the possibility of getting on-site HCW segregation containers at waste generation point seems to be a positive factor that motivates healthcare workers to segregate HCW. In support of this assertion, studies showed the positive correlation between good HCW segregation practice and presence of on-site color-coded waste collection container [27, 30, 32].

In the present study, almost two-thirds (65%) of the respondent had awareness of HCW segregation. This finding is lower than a study report from Uganda, 71.8% [32], and Portugal [45]. This can be attributed to differences in study participants, setting and compliance toward HCWM recommendations.

On the other hand, it was found that the odds of good HCW segregation practice were 1.7 times more likely among male healthcare workers than female healthcare workers. The possible explanation for this finding might be linked with the standard precaution practice and attitude of male healthcare workers. In this study, the majority of male healthcare workers had good self-reported standard precaution practice and positive attitude towards HCW segregation than female’s healthcare which may increase male HCW segregation practices.

Age of healthcare worker was another socio-demographic factor that significantly associated with self-reported HCW segregation practice. Those young age study participants were about two time’s higher odds of good self-reported HCW segregation practice. Although the reason why younger healthcare workers were more likely to had good HCW segregation practice than their counterparts is beyond the scope of this study, the probable reason is that, in this study younger healthcare workers tend to have less work experience, which is associated with higher odds of self-reported HCW segregation practice as evidence from multivariable logistic regression analysis. In line with this study, a study by Mesfin et al. also reported similar finding [30].

In this study, physicians and health officers were 55% less likely to have good self-reported HCW segregation practice than nurses and midwives. On the contrary, one study from Gondar (north Ethiopia) indicates that nurses were 73% less likely to have correct HCW segregation practice than physicians [30]. The possible reason for this inconsistency could be a difference in measurement of the construct items and study facilities; in the previous study, a single hospital was included as opposed to this study which includes five hospitals. In addition, laboratory technicians and technologist were 2.8 times more likely to have good self-reported HCW segregation practice than nurses and midwives. In addition, healthcare workers who had good self-reported standard precaution practice were eight times more likely to have good self-reported HCW segregation practice than their counterparts.

This study had several limitations that need to be considered when interpreting the results. First, the estimated compliance of HCWs segregation practice and their associated factors may be subject to reporting errors, because all the information came from the self-reports of the survey participants. Second, social desirability bias may have been present in the form of the over-reporting of HCW segregation compliance in the survey. Third, the cross-sectional design of the survey did not allow any conclusion in terms of a specific causal direction. Fourth, the reader needs to take precautionary measure while interpreting the study finding since general hospital seems to provide most of the data. One additional limitation of the study is that the data collection tool used in the present study is not validated. Lack of validated questionnaires with acceptable reliability and validity for assessing healthcare waste segregation practice in Ethiopia was the major limitation of the study that limits the present findings. To overcome this problem the study included items that are acceptable face-validly and reliability.

## Conclusion

Overall, only half of the healthcare workers had good healthcare waste segregation practice, which is low and unsatisfactory. Less service year, having good standard precaution practice, and the presence of onsite waste segregation container were the most important variables that correlate with self-reported healthcare waste segregation practice. In order to improve HCW segregation practice, health authorities should consider those identified factors. In addition, continuous mentorship and supervision on HCWM were recommended at all level to safeguard healthcare workers, patients and community from impending consequences as a result of inadequate segregation and indiscriminate disposal of sharp HCW. Moreover, health authorities should focus on sufficient allocation of onsite waste receptacles and on periodic training towards standard precaution will improve compliance with segregation practice. Further study also needed to determine the healthcare workers actual segregation practice.

## Additional files

Additional file 1: The schematic presentation of sampling procedure (PDF 301 kb)

Additional file 2: Standard precaution practice score (PDF 184 kb)

Additional file 3 Data collection tool (PDF 409 kb)

## Data Availability

Data will be available upon request from the corresponding authors.

## Abbreviations

AOR: Adjusted odds ratio
CI: Confidence interval
COR: Crude Odds ratio
HBV: Hepatitis B virus
HCFs: Healthcare facilities
HCV: Hepatitis C virus
HCW: Healthcare wastes
HCWM: Healthcare waste management
HIV: Human Immunodeficiency virus
SOP: Standard Operating Procedure
SPSS: Statistical Package for Social Sciences
WHO: World Health Organization
