# Supplementary figures and images for: Self-reported healthcare waste segregation practice and its correlate among healthcare workers in hospitals of Southeast Ethiopia
Source: BMC Health Serv Res. 2019 Aug 22;19:591. doi: 10.1186/s12913-019-4439-9 (PMC6704682; doi:10.1186/s12913-019-4439-9)

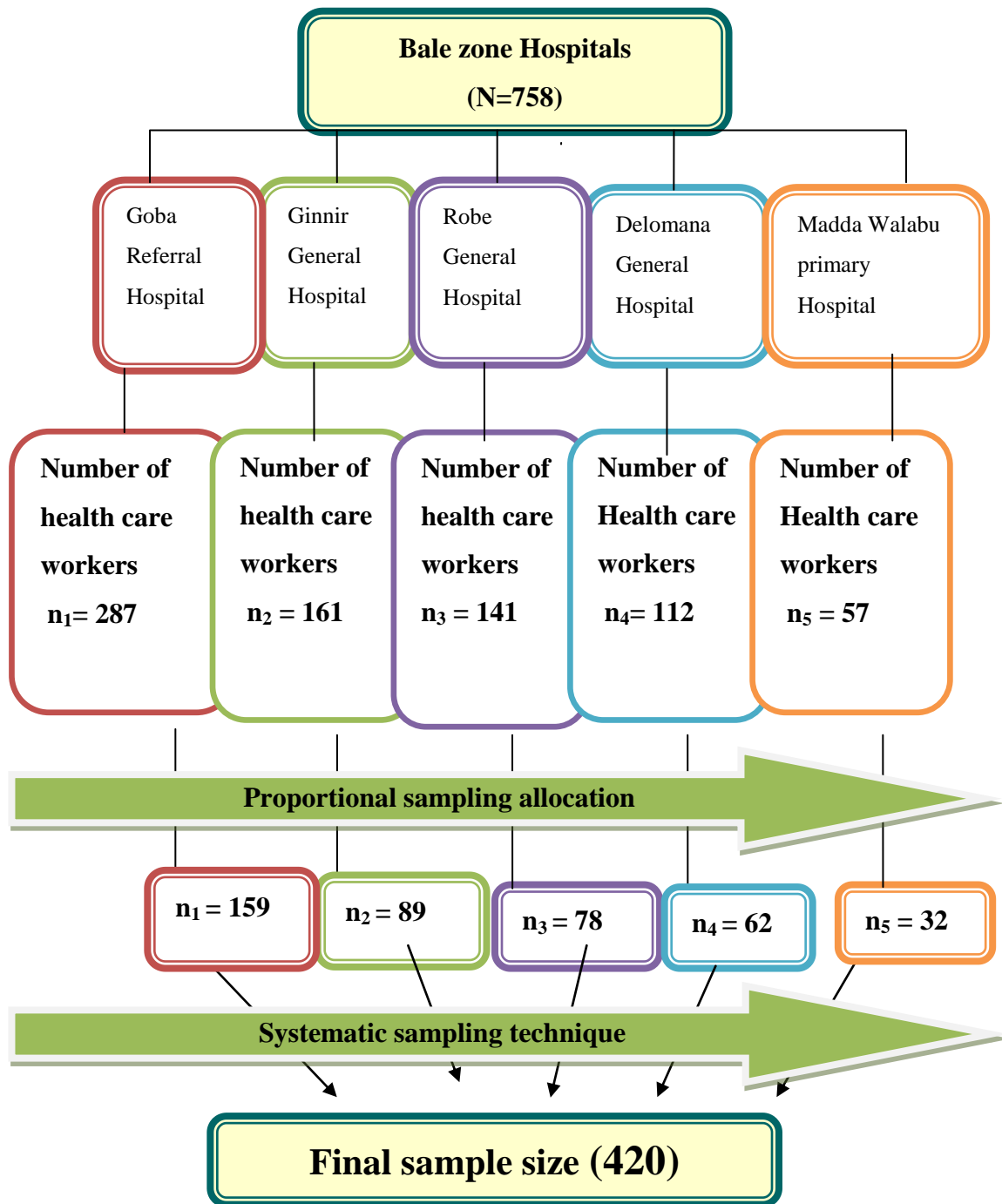

Figure 1: Schematic presentation of sampling procedure

Supplement: Supplementary file 1 — The schematic presentation of sampling procedure (PDF 301 kb) [file 12913_2019_4439_MOESM1_ESM.pdf]
