# Supplementary material for: Self-reported healthcare waste segregation practice and its correlate among healthcare workers in hospitals of Southeast Ethiopia
Source: BMC Health Serv Res. 2019 Aug 22;19:591. doi: 10.1186/s12913-019-4439-9 (PMC6704682; doi:10.1186/s12913-019-4439-9)
Supplement: Supplementary file 2 — Standard precaution practice score (PDF 184 kb) [file 12913_2019_4439_MOESM2_ESM.pdf]

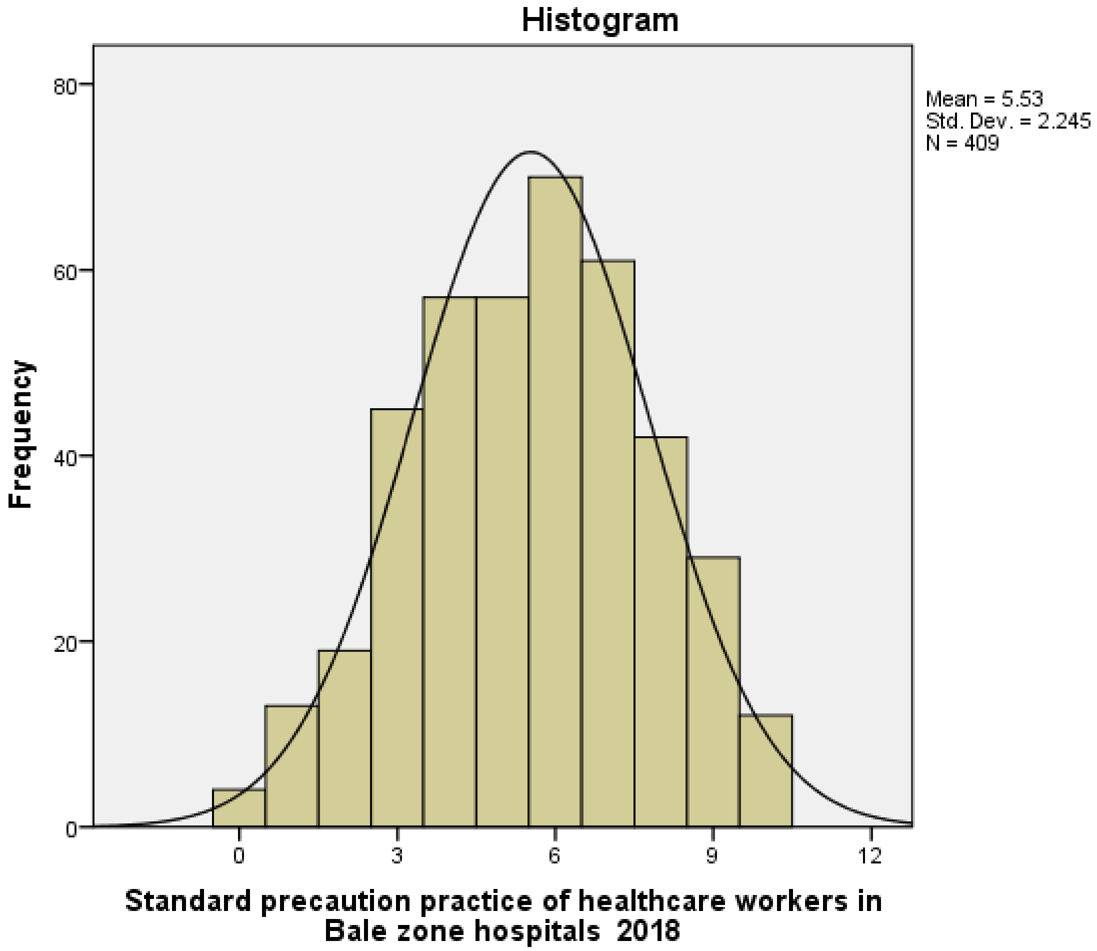

Figure 1: Standard precaution practice composite score distribution of healthcare workers in Bale zone, Southeast Ethiopia 2018.
