# Supplementary material for: Self-reported healthcare waste segregation practice and its correlate among healthcare workers in hospitals of Southeast Ethiopia
Source: BMC Health Serv Res. 2019 Aug 22;19:591. doi: 10.1186/s12913-019-4439-9 (PMC6704682; doi:10.1186/s12913-019-4439-9)
Supplement: Supplementary file 3 — Data collection tool (PDF 409 kb) [file 12913_2019_4439_MOESM3_ESM.pdf]

<u>Data Collection Tool</u>

PART ONE: 1.1 SOCIO-DEMOGRAPHIC DATA

S.No	Item/Questions	Response option	Code	Skip pattern
101	Hospital level	[ ] Referral hospital	1	
		[ ] General hospital	2	
		[ ] Primary hospital	3	
102	Current working department	[ ] Internal medicine	1	
		[ ] Surgical ward	2	
		[ ] Pediatrics ward	3	
		[ ] Neonatal intensive care unit (NICU)	4	
		[ ] Obstetrics and Gynecology	5	
		[ ] Operating room (OR)	6	
		[ ] Emergency-unit	7	
		[ ] Outpatient department (OPD)	8	
		[ ] Laboratory	9	
		[ ] Other (specify: _______	10	
103	Sex (*do not ask*)	[ ] Male	1	
		[ ] Female	2	
104	What is your Profession?	[ ] Doctor	1	
		[ ] Nurse/ Midwife	2	
		[ ] Health officer	3	
		[ ] Laboratory technicians and technologist	4	
		[ ] Other (specficy):__________	5	
105	What is your Educational status?	[ ] Diploma	1	
		[ ] 1st degree	2	
		[ ] 2nd degree and above	3	
106	What is your current age?	( ) years		
107	Years of service after the last graduatio n?	( ) years		

108	What is your Marital status?	[ ] Single	1	
		[ ] Married	2	
		[ ] Divorce/ separated	3	
		[ ] Widowed	4	

PART ONE: - 1.2 GENERAL INFORMATION ON HCWs SEGRGATION

S.No	Item/Questions	Response option	Code	Skip pattern
109	Ever taking training in HCWM methods	[ ] Yes	1	
		[ ] No	2	
110	Presence of guideline, SOP or instructive poster on HCW segregation in your working area?	[ ] Yes	1	
		[ ] No	2	
111	Do you think HCW segregation practice is a key and first step in safe HCWM process?	[ ] Yes	1	
		[ ] No	2	
112	Do you have on-site HCW segregation containers in your working area ?	[ ] Yes	1	
		[ ] No	2	
113	What are the different categories of HCWs ? (***multip le answer possible)***	[ ] General wastes	1	
		[ ] Sharps	2	
		[ ] Infectious wastes	3	
		[ ] Pathological wastes	4	
		[ ] Pharmaceutical waste	5	
		[ ] Chemical wastes	6	

PART TWO: - SP PRACTICES OF HCWS

S.no	Item/Questions	Response option	Code	Skip pattern
201	Do you wash your hand before and after patient contact?	[ ] Yes	2	
		[ ] No	1	
202	Do you wear utility gloves while handling, contaminated it ems?	[ ] Yes	2	
		[ ] No	1	
203	Do you wear facemask or face shield when undertaking pr ocedures likely to generate splashes on to eye or mouth ?	[ ] Yes	2	
		[ ] No	1	
204	Do you wear apron during procedures and/or patient care activities when contact of clothing or exposed skin with bl ood/body fluids, secretions, and excretions is anticipated?	[ ] Yes	2	
		[ ] No	1	
205	Do you avoid recapping, bending, breaking and hand-man	[ ] Yes	2	

	ipulating used needles	[ ] No	1	
206	Do you immediately decontamination surgical instruments or critical items or any reusable items in 0.5 % decontami nant solution?	[ ] Yes	2	
		[ ] No	1	
207	Do you check and verify before administering injection it is given to the right person, the right drug, the right dose, th e right time, the right route and the right injection equipme nt?	[ ] Yes	2	
		[ ] No	1	
208	Do you use Sterile syringes and sterile needles while adm inistering all medication (Intramuscular, Intravenous and I ntradermal) or collect blood from patients?	[ ] Yes	2	
		[ ] No	1	
209	Do you wear the necessary personal protective equipmen ts like mask during tuberculosis suspected patient examin ation	[ ] Yes	2	
		[ ] No	1	
210	Do you use antiseptic agents for cleansing the skin or mu cous membrane prior to invasive procedures/surgery, clening wounds, or performing handrubs or surgical hand scrubs using an alcohol based antiseptic products	[ ] Yes	2	
		[ ] No	1	

PART THREE: - ATTITUDE TOWARDS HCWS SEGREGATION PRACTICE

Sr.no	Attitude related questions	Response	Code	Skip pattern
301	Segregation of HCWs should be done at the point of gene ration ?	[ ] Agree	1	
		[ ] Disagree	2	
		[ ] Neutral	3	
302	The color-coding system aims at ensuring immediate iden tification and segregation of the hazards associated with t he type of HCWs	[ ] Agree	1	
		[ ] Disagree	2	
		[ ] Neutral	3	
303	Waste should be separated by the person generating the waste immediately according to its type?	[ ] Agree	1	
		[ ] Disagree	2	
		[ ] Neutral	3	
304	Non appropriate segregation of HCWs may leads to occu pational hazard in healthcare facilities?	[ ] Agree	1	
		[ ] Disagree	2	
		[ ] Neutral	3	
305	Syringes and needles should be discarded without recapp ing.	[ ] Agree	1	
		[ ] Disagree	2	
		[ ] Neutral	3	

306	Waste handlers should never sort through waste after it h as been placed in the bin.	[ ] Agree	1	
		[ ] Disagree	2	
		[ ] Neutral	3	

PART FOUR: - HCWs SEGREGATION PRACTICE ITEMS

S.No	Item/Questions	Response option	Code	Skip pattern/r emark
401	Do you segregate HCWs for the last client attended	[ ] Yes	1	
		[ ] No	2	**If "No" go to Q 402**
401.1	*If "**YES**" ask the healthcare workers to describe the type of wastes that was discarded ?*<br>*/ **NOTE**/: lavel if only corresponded with correct segregation practices as sharps, infectious, non-infec tious, pharmaceuticals, pathologic & chemicals*	____________<br>____________<br>____________<br>____________<br>____________<br>____________	1<br>2<br>3<br>4<br>5<br>6	
402	Do you perform segregation at wastes generation point ?	[ ] Yes	1	
		[ ] No	2	
403	Do you segregate syringes and needles without recapping or bending and immdiately put them in pucture resistant s afety box?	[ ] Yes	1	
		[ ] No	2	
404	*Do you always segregate infectious wastes such as blood ,blood products & other body fluids or items contaminated with similar fluids ?*	[ ] Yes	1	
		[ ] No	2	
405	*Do you separate items contaminated by or containing pha rmaceutical products or outdated medications and residua ls of drugs ?*	[ ] Yes	1	
		[ ] No	2	
406	*Do you segregate general wastes such as paper, trash, boxes, bottles, plastic containers, leftover foods and food products from the other HCWs*	[ ] Yes	1	
		[ ] No	2	
407	Do separately handle/segregate chemical wastes such as formaldehyde, disinfectant products, solvents, organic & inorganic chemicals	[ ] Yes	1	
		[ ] No	2	
408	Do you segregate placenta/ human tissues, body parts an d fetus in different container and disposed accordingly	[ ] Yes	1	
		[ ] No	2	
409	Do you disinfect /decontaminate sharp wastes before	[ ] Yes	1	

	disposal	[ ] No	2	
410	Do you decontaminate infectious wastes before disposal	[ ] Yes	1	
		[ ] No	2	
411	Do you place safety boxes at hand reached area	[ ] Yes	1	
		[ ] No	2	
